# Polygenic risk score for hypercholesterolemia in a Brazilian familial hypercholesterolemia cohort

**DOI:** 10.1016/j.athplu.2022.06.002

**Published:** 2022-06-28

**Authors:** Isabella Ramos Lima, Mauricio Teruo Tada, Theo G.M. Oliveira, Cinthia Elim Jannes, Isabela Bensenor, Paulo A. Lotufo, Raul D. Santos, Jose E. Krieger, Alexandre C. Pereira

**Affiliations:** a Laboratory of Genetics and Molecular Cardiology, Heart Institute (InCor), University of São Paulo Medical School, São Paulo, Brazil; b Center for Clinical and Epidemiologic Research, University of São Paulo, São Paulo, Brazil; c Lipid Clinic, Heart Institute (InCor), University of São Paulo Medical School Hospital, São Paulo, Brazil; d Genetics Department, Harvard Medical School, Boston, MA, USA

**Keywords:** Polygenic risk score, Familial hypercholesterolemia, Genetics, Lipid disorder, Atherosclerosis

## Abstract

**Background and aims:**

Familial hypercholesterolemia (FH) is a genetic disorder characterized by high levels of LDL-C leading to premature cardiovascular disease (CAD). Only about 40% of individuals with a clinical diagnosis of FH have a causative genetic variant identified, and a proportion of genetically negative cases may have a polygenic cause rather than a still unidentified monogenic cause. This work aims to evaluate and validate the role of a polygenic risk score (PRS) associated with hypercholesterolemia in a Brazilian FH cohort and its clinical implications.

**Methods:**

We analyzed a previously derived PRS of 12 and 6 SNPs (Single Nucleotide Polymorphism) in 684 FH individuals (491 mutation-negative [FH/M−], 193 mutation-positive [FH/M+]) and in 1605 controls. Coronary artery calcium (CAC) score was also evaluated.

**Results:**

The PRS was independently associated with LDL-C in control individuals (*p* < 0.001). Within this group, in individuals in the highest quartile of the 12 SNPs PRS, the odds ratio for CAC score >100 was 1.7 (95% CI: 1.01–2.88, *p* = 0.04) after adjustment for age and sex. Subjects in the FH/M− group had the highest mean score in both 12 and 6 SNPs PRS (38.25 and 27.82, respectively) when compared to the other two groups (*p* = 2.2 × 10-16). Both scores were also higher in the FH/M+ group (36.48 and 26.26, respectively) when compared to the control group (p < 0.001 for the two scores) but inferior to the FH/M− group. Within FH individuals, the presence of a higher PRS score was not associated with LDL-C levels or with CAD risk.

**Conclusion:**

A higher PRS is associated with significantly higher levels of LDL-C and it is independently associated with higher CAC in the Brazilian general population. A polygenic cause can explain a fraction of FH/M− individuals but does not appear to be a modulator of the clinical phenotype among FH individuals, regardless of mutation status.

## Highlights

• Pathogenic variants are not detected in ∼60% of FH individuals, and part of these cases may have a polygenic cause.
• Patients without a monogenic cause of FH may present a specific combination of SNPs that, together, elevate the LDL-C levels.
• This is the first study that shows the applicability of a dyslipidemia PRS in Latin American populations.
• Subjects with FH without a causal genetic variant had a higher mean PRS compared to FH mutation-positive and control groups.
• Despite not predicting CVD events, the PRS is a risk predictor of preclinical atherosclerosis in the general population.

## 1 Introduction

Familial hypercholesterolemia (FH) is an autosomal dominant genetic disorder characterized by high levels of low-density cholesterol (LDL-C) leading to premature coronary heart disease. It is estimated that heterozygous FH affects about 1 in 300 individuals worldwide, while the homozygous form affects around 1 in 300.000 [1,2]. A study by Harada et al. estimated that the prevalence of FH in the Brazilian population is around 1 in 263 individuals [3].

FH is mainly caused by genetic defects in the *LDLR* gene [4], where 93% of the causative genetic variants are located. Another 5% of causal FH genetic variants are in the *APOB* gene [5,6]. In addition, 2% of the genetic variants are gain-of-function variants in the *PCSK9* gene, responsible for encoding the subtilisin/kexin type 9 pro-protein convertase [4,7].

Despite the number of patients diagnosed with the FH phenotype has increased in recent years, pathogenic genetic variants are not detected in about 60% of clinically diagnosed individuals [8]. Although this number depends on the defined inclusion criteria and the investigated genes, it has been proposed that a proportion of the negative cases may have a polygenic cause, rather than a still unidentified monogenic cause [9]. In fact, the realization of the great inter-individual heterogeneity of both the clinical presentation and prognosis of FH patients, even when carrying the same pathogenic variant, highlights the importance of understanding sources of residual variation in FH patients and hyperlipidemia.

It is assumed that in polygenic hypercholesterolemia, patients who does not have an identified monogenic cause of FH present a specific combination of common (single nucleotide variations) SNPs that, together, elevate the LDL-c concentrations, exceeding the diagnostic threshold. Polygenic hypercholesterolemia could explain the disparity between the individuals who present a molecular diagnosis and individuals who have only a clinical diagnosis without any pathogenic genetic variant identified [10].

Talmud et al. [10] proposed a polygenic risk score (PRS) of 12 SNPs derived from the weighted sum of the number of risk alleles found in each individual. The weights used are the β-coefficients of each risk allele reported on a lipid GWAS meta-analysis performed by the Global Lipid Genetics Consortium (GLGC) [11]. In a complementary study, the group later showed that this score could be efficiently decreased from 12 to 6 SNPs [12,13]. Currently, the replication of this PRS has already been carried out in at least 6 European countries, in Israel and Korea, and in all studies, the score was significantly associated with LDL-c values [13,14]. In addition, a recent study showed this score distribution and association with LDL-C in three different UK ethnic groups [15].

However, most analyses with this PRS were performed on European or Asian individuals. To the best of our knowledge, up to this date, no study has shown the applicability of PRS in Latin American populations which may have a diverse ancestry and genetic backgrounds. In addition, although the association structure between PRS and lipid-related phenotypes has been replicated in the general population [16,17], its applicability in individuals with FH is less clear. Therefore, we aim to test whether the PRS scores of 12 and 6 SNPs apply to Brazilian FH cases and what are their clinical implications. For that we have tested the association of PRS with cholesterol levels and preclinical and clinical manifestations of atherosclerosis in individuals that had been submitted to genetic FH cascade screening and in a subgroup of participants of the ELSA-Brasil study.

## 2 Materials and methods

### 2.1 Subjects

We analyzed 684 non-related adults included in the HipercolBrasil, the largest FH genetic cascade screening program in Latin America with around 5000 patients enrolled [18], conducted at the Laboratory of Genetics and Molecular Cardiology at the Heart Institute (InCor), University of São Paulo Medical School, São Paulo, Brazil. The study was conducted in accordance with the Declaration of Helsinki and was approved by the local institutional review boards (Ethics Committee CAPPesq number 3757/12/013). All subjects signed an informed consent form. HipercolBrasil program inclusion criteria were LDL-C ≥210 mg/dL, and exclusion criteria were triglycerides ≥400 mg/dL, liver failure, nephropathy, uncontrolled hypothyroidism, and infection by the HIV [18].

As a comparative group, we analyzed 1605 healthy adults from the ELSA-Brasil (Brazilian Longitudinal Study of Adult Health) study, a multicenter cohort composed of individuals aged between 35 and 74 years old from six public research institutions in different regions of Brazil. The main objective of the ELSA-Brasil is to investigate the incidence, progressions, and risk factors for chronic diseases, particularly cardiovascular diseases and diabetes [19]. For the present analysis, we have only used participants from the ELSA-Brasil Sao Paulo site, the same city in which HipercolBrasil has ascertained FH cases.

### 2.2 Genetic sequencing

Next-generation sequencing (NGS) was performed in all selected subjects in two different ways. Four hundred forty samples were sequenced using an AmpliSeq panel on Ion Torrent PGM platform (Thermo Fisher) that included *LDLR*, *APOB*, *PCSK9*, *LDLRAP1,* and *LIPA* genes and 10 of the 12 SNPs presented in the PRS. The two remaining SNPs were genotyped using a TaqMan assay (Thermo Fisher) in a QuantStudio 12K Flex Real-Time PCR System.

The other 244 samples were sequenced through the SureSelect QXT panel by Agilent Technologies, including all genes of the AmpliSeq panel plus the *ABCG5*, *ABCG8*, *APOE,* and *STAP1* genes and the 12 SNPs of the PRS.

In participants where no genetic variants associated with FH were found, MLPA (MRC-Holland) was performed to screen for the presence of copy number variations in the *LDLR* gene.

Population databases GnomAD and ABraOm (Brazilian Online Mutation Database) were used to assess the frequency of variants. *In silico* predictions were also verified, using SIFT, PolyPhen-2, and PROVEAN algorithms. HGMD and ClinVar literature databases were consulted. Variant classification was made according to the ACMG recommendations.

The group that had no identified causal variant was classified as FH/M− (n = 491) and the group in which a unique pathogenic or likely-pathogenic variant was found was classified as FH/M+ (n = 193). Variants of uncertain significance (VUS) were not considered in this study.

Control subjects were genotyped through Axiom™ Precision Medicine Research Array (Thermo Fisher) using the GeneTitan MultiChannel system, following manufacturer's instructions.

### 2.3 Polygenic risk score calculation and analysis

The polygenic risk score was performed for each FH group (FH/M− and FH/M+) and in the control group (ELSA-Brasil). The score was calculated with a weighted sum of the risk alleles (the allele associated with increased LDL-C levels) where the weights used were the β-coefficient of each SNP reported by the GLGC [11]. In all groups, the PRS was performed with both 12 and 6 SNPs.

For the LDL-C distribution analysis among the scores, we divided it into tertiles. If available, baseline LDL-C was used, and for statins and/or ezetimibe users we adjusted the LDL-C concentration multiplying the LDL-C values by the coefficient correction 1.43, as previously described [20]. To compare the occurrence of CVD with the value of PRSs in each group, the scores were divided into quartiles.

To investigate the response to pharmacological treatment according to the PRS, we analyzed the LDL-C reduction in individuals in the FH groups through the difference between the LDL-C value at baseline (no treatment) and after pharmacological treatment with statins and/or ezetimibe.

### 2.4 Coronary artery calcium (CAC) measurement

A CAC score was available in 1497 subjects of the control group, 81 in the FH/M− group, and 32 subjects in the FH/M+. Scans were performed on a 64-slice multi-detector computed tomography (MDCT) scanner (Philips Brilliance; Philips, Netherlands), with a standard technique for CAC scoring, including prospective acquisition in mid-diastole; 120 KVp tube voltage; and variable current based on body mass index. CAC was measured according to the Agatston method of area-density product summation. The total CAC score was obtained by adding the individual lesion area-density products from all epicardial coronary arteries. This method is both reliable and reproducible, with low interscan variability [21].

### 2.5 Statistical analysis

Statistical analyses were performed using R 3.6.1. An initial descriptive analysis was carried out in the groups. Categorical variables are described as frequencies. To compare the difference between those, we used the Chi-square test. For quantitative variables with non-normal distribution, median and first and third interquartile range were calculated, and data analyzed with Mann–Whitney *U* test to compare the medians. For comparison of more than two groups, Kruskall Wallis test was used, with multiple post-hoc test comparison.

Linear regression was used to assess the association between LDL-C levels and polygenic scores among the groups and control subjects. Statistical significance was considered at a *p*-value <0.05.

### 2.6 Patients and public involvement

Patients and public were not involved in the design, conduct and concept of our study.

## 3 Results

### 3.1 Subjects characteristics

Six hundred and eighty-four individuals with a clinical diagnosis of FH were analyzed. One hundred and ninety-three subjects were in the FH/M+ group and 491 individuals were in the FH/M− group. The clinical and laboratory characteristics of these subjects and the 1605 individuals in the control group are listed in Table 1. FH/M− patients had a higher proportion of women and older individuals when compared to the other two groups (*p* < 0.001). Significant differences were also observed in body mass index (*p* = 0.01) and all the measured laboratory values between groups (*p* < 0.001). LDL-C values without lipid-lowering treatment (baseline) was available in 466 individuals of FH groups. Among these individuals, the positive group had a higher frequency of pharmacological treatment at enrolment when compared with the individuals in the HF/M− group (*p* = 0.004), and there was a higher frequency of previous MI in the FH/M+ group (*p* = 0.01), but no statistical differences were observed regarding stroke and myocardial revascularization (Table 1).

**Table 1:** Characteristics of patients included in the study.

Variables	ELSA-Brasil (n = 1605)	FH/M-(n = 491)	FH/M+ (n = 193)	*p*-value
**Age (years)**	50 (45–57)	56 (46–63)	47 (36–58)	**<0.001**
**Female sex, n (%)**	868 (54.1)	336 (68.4)	116 (60.10)	**<0.001**
**Self-declared ancestry**				
White, n (%)	959 (59.75)	274 (55.80)	115 (59.58)	**0.045**
Mixed, n (%)	386 (24.05)	87 (17.72)	37 (19.17)	
Black, n (%)	211 (13.15)	39 (7.94)	10 (5.18)	
Indigenous, n (%)	19 (1.18)	7 (1.43)	2 (1.04)	
Asian, n (%)	13 (0.81)	2 (0.41)	2 (1.04)	
Others or nondeclared, n (%)	17 (1.06)	82 (16.70)	27 (13.99)	
**Medical history**				
Myocardial infarction, n (%)	–	75 (15.27)	46 (23.83)	**0.011**
Stroke, n (%)	–	21 (4.28)	9 (4.66)	0.988
Myocardial revascularization n (%)	–	25 (5.09)	17 (8.80)	0.099
Hypertension, n (%)	515 (32.09)	216 (43.99)	55 (28.50)	**<0.001**

**Smoking habit**				
Current smoker, n (%)	259 (16.13)	65 (13.23)	24 (12.44)	**0.002**
Former smoker, n (%)	489 (30.47)	143 (29.12)	37 (19.17)	
Never smoke, n (%)	857 (53.40)	276 (56.21)	130 (67.36)	
**Laboratory values**				
Total Cholesterol (mg/dL)	208 (185–236)	323.5 (305–346)	341.5 (318–394)	**<0.001**
Triglycerides (mg/dL)	116 (80–164)	156 (114–210)	137.5 (100.5–178.2)	**<0.001**
HDL-C (mg/dL)	53 (46–63)	50 (42–59.25)	47 (39–55)	**<0.001**
LDL-C without lipid-lowering therapy	127 (106–150)	239 (223–258)	277 (244–325)	**<0.001**
LDL-C with lipid-lowering therapy		116.5 (94–142)	142 (111–177)	**<0.001**

**Therapies at enrolment∗∗**				
Lipid-lowering therapy, n (%)		343 (71.45)	158 (82.29)	**0.004**
Antiplatelet, n (%)		128 (26.07)	60 (31.09)	0.232
Antihypertensives, n (%)		128 (26.07)	56 (29.01)	0.065
**Physical examination**				
BMI	26.70 (24.01–29.76)	27.28 (24.28–30.85)	26.73 (23.80–30.71)	**0.015**
Xanthoma, n (%)		10 (2.04)	23 (11.92)	**<0.001**
Xanthelasma, n (%)		40 (8.15)	23 (11.92)	0.162
Corneal arcus, n (%)		5 (1.02)	5 (2.59)	0.226
**Dutch Lipid Clinic Network Score (DLCNS)**				
Definite FH, n (%)		44 (8.96)	66 (34.20)	**<0.001**
Probable FH, n (%)		162 (32.99)	75 (38.86)	
Possible FH, n (%)		255 (51.94)	39 (20.21)	
Missing information, n (%)		30 (6.11)	13 (6.73)	

Abbreviations: BMI – body mass index (kg/m^2^); FH – Familial hypercholesterolemia.

Continuous variables with abnormal distribution are expressed as median and quartiles 1 and 3 (Age, Total cholesterol, Triglycerides, HDL-cholesterol, LDL-cholesterol, and BMI). Mann–Whitney *U* test was used to compare the medians between two groups and Kruskall Wallis test was used for three groups comparison.

Categorical variables (Sex, Ancestry, Medical History, Pharmacological Treatment, Smoking Habit, Xanthoma, Xanthelasma, Corneal Arcus and Dutch Lipid Clinic Network Score) are expressed as absolute numbers and percentages. To compare the difference between those variables, we used the Chi-square test.∗∗ For lipid-lowering therapy, statins and ezetimibe were considered. ASA (acetylsalicylic acid) and clopidogrel were considered antiplatelet drugs. ACE inhibitors, angiotensin receptor blockers, diuretics, and alpha-blockers were considered antihypertensives.

### 3.2 Polygenic risk score

**Fig. 1:**
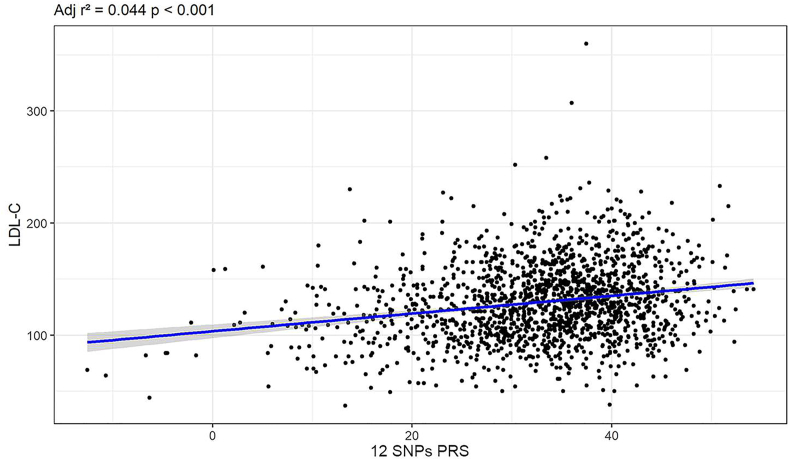
Association between 12 SNPs PRS and LDL-C in individuals from the ELSA-Brasil group.

The 12 and 6 SNPs present in the PRS and their respective weights and frequencies are described in Table 2. As a result of simple linear regression and Pearson correlation performed in the control group, we observed a significant association between LDL-C and both scores: 12 SNPs PRS: Adjusted R^2^ = 0.044 *p* < 0.001 (Fig. 1) and 6 SNPs PRS: Adjusted R^2^ = 0.046, *p* < 0.001 (Supplementary Figure 1). We did not observe significant differences regarding the effect size of the derived PRS and LDL-C among the different self-referred ancestry in the Brazilian population in the 12 SNPs PRS. However, in the third tertile of the 6 SNPs PRS, we observed a significant difference between White and Black groups (Supplementary Table 1).

**Table 2:** List of genotyped SNPs associated with an increased LDL-c.

SNP	Gene	Minor Allele	Common Allele	GLGC weight	Minor Allele Frequency			6 SNPs PRS
					ELSA-Brasil (n = 1605)	FH/M+ (n = 193)	FH/M-(n = 491)	
rs2479409	*PCSK9*	***G***	*A*	2.01	0.358	0.388	0.400	
rs629301	*CELRS2*	*G*	***T***	5.65	0.244	0.166	0.180	Yes
rs1367117	*APOB*	***A***	*G*	4.05	0.241	0.285	0.317	Yes
rs4299376	*ABCG8*	***G***	*T*	2.75	0.271	0.290	0.311	Yes
rs1564348	*SLC22A1*	*C*	***T***	0.56	0.163	0.184	0.178	
rs1800562	*HFE*	*A*	***G***	2.22	0.020	0.020	0.014	
rs3757354	*MYLIP*	*T*	**C**	1.43	0.246	0.280	0.224	
rs11220462	*ST3GAL4*	***A***	*G*	1.95	0.101	0.109	0.120	
rs8017377	*NYNRIN*	***A***	*G*	1.14	0.348	0.399	0.373	
rs6511720	*LDLR*	*T*	***G***	6.99	0.134	0.111	0.058	Yes
rs429358	*APOE*	*C*	*T*	–	0.139	0.153	0.215	Yes
rs7412	*APOE*	*T*	*C*	–	0.070	0.023	0.018	Yes
ϵ2ϵ2	*APOE*			−34.75	0.007	0	0.008	
ϵ2ϵ3	*APOE*			−15.45	0.107	0.041	0.012	
ϵ2ϵ4	*APOE*			−7.72	0.02	0.005	0.004	
ϵ3ϵ3	*APOE*			0	0.624	0.601	0.556	
ϵ3ϵ4	*APOE*			3.86	0.225	0.207	0.305	
ϵ4ϵ4	*APOE*			7.72	0.017	0.031	0.045	

Abbreviations: SNP - Single Nucleotide Polymorphism; GLGC - Global Lipid Genetic Consortium; Minor Allele Frequency (MAF).

Risk Alleles are in bold. *APOE* weights were based on haplotypic effects. The weights reported by GLGC are in mg/dL.

**Fig. 2:**
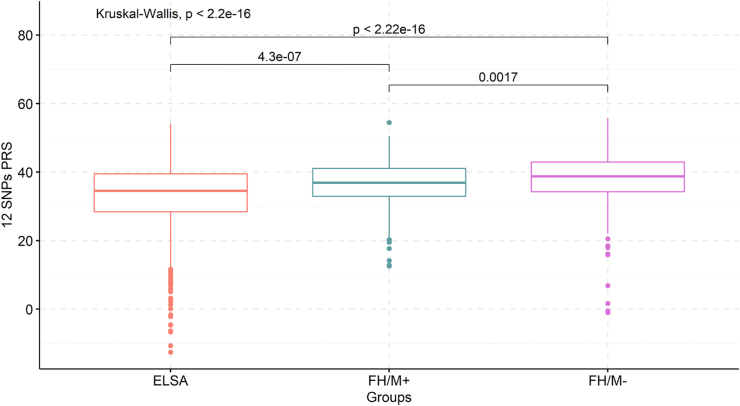
Comparison of mean value 12 SNPs PRS between ELSA-Brasil, FH/M+ and FH/M − groups.

Once showing that the scores were associated with LDL-C in the general Brazilian population, we verified how the scores differ between the studied groups. The control group had the lowest mean score in both 12 SNPs (33.10 [SD 9.13]) and 6 SNPs PRS (23.03 [SD 8.80]) when compared to the FH groups. The FH/M− group had the highest mean in both weighted scores (12 SNPs PRS mean 38.25 [SD 7.23] and 6 SNPs PRS mean 27.82 [SD 6.87]) followed by the FH/M+ group (12 SNPs PRS mean 36.48 [SD 6.97] and 6 SNPs PRS mean 26.26 [SD 6.66]) (Supplementary Table 2). When compared, the difference between all the groups was statistically significant (*p* < 0.001 in both scores). Fig. 2 shows those comparisons in the 12 SNPs PRS (See Supplementary Figure 2 for results from the 6 SNPs PRS).

**Fig. 3:**
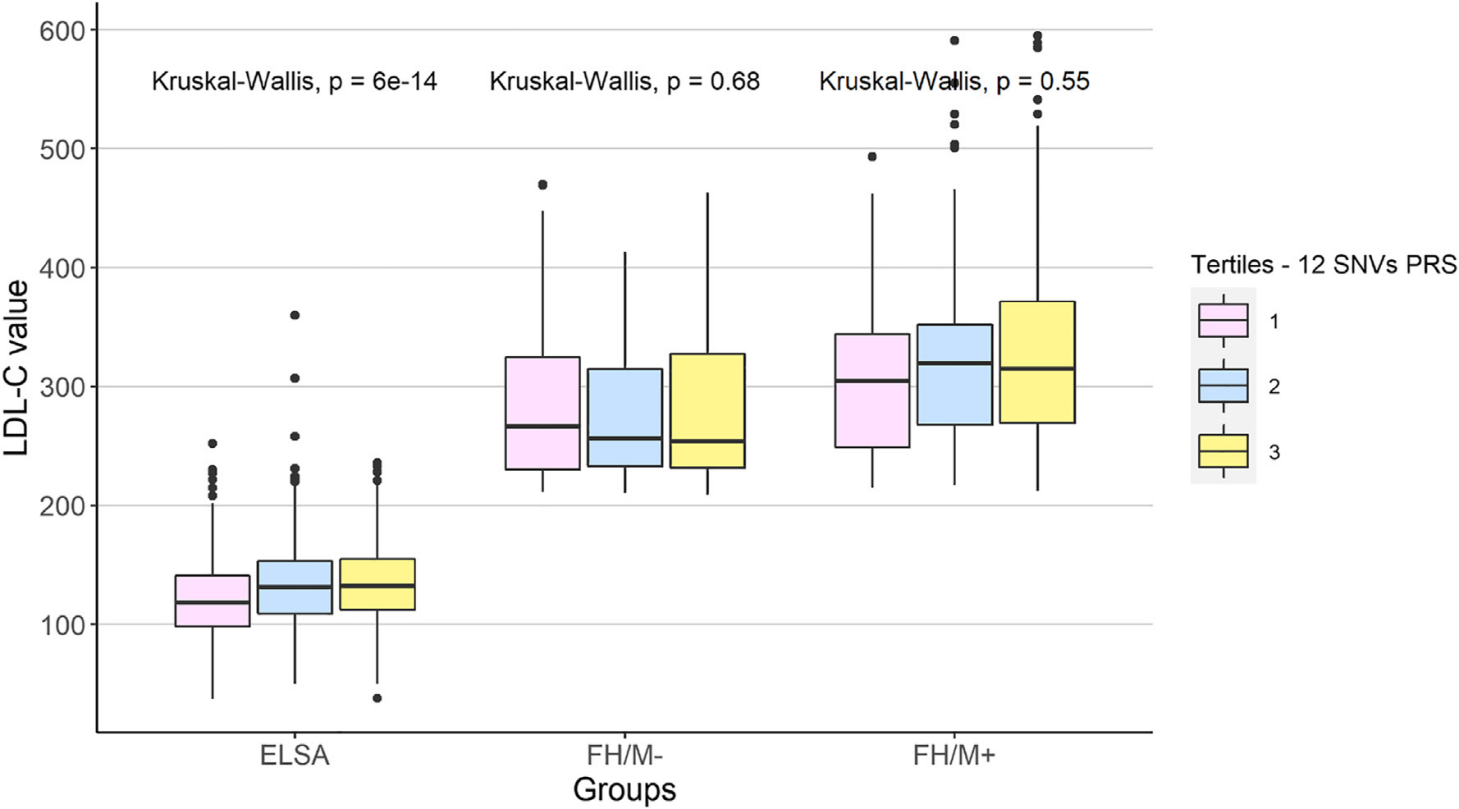
Distribution of LDL-C value between tertiles of 12 SNPs PRSs in each study group.

As shown in Fig. 3, patients in the highest tertile of the 12 SNPs PRS in the control group had a higher LDL-C value when compared to the lowest tertiles (*p* < 0.001 in the two scores). However, this difference was not observed in the FH groups. In both FH/M− and FH/M+ individuals, the LDL-C value was not statistically different between the tertiles of both scores (FH/M-: 12 SNPs PRS *p* = 0.68; FH/M+: 12 SNPs PRS *p* = 0.55) See Supplementary Figure 3 to 6 SNPs PRS.

As a statistically significant difference was observed in terms of ancestry between groups, we performed a sub-analysis, dividing the individuals into whites and non-whites. Similarly, both white and non-white individuals in the FH group had a higher mean score when compared to the control group (Supplementary Table 3, Supplementary Figure 4).

### 3.3 Response to pharmacological treatment in the FH group

**Fig. 4:**
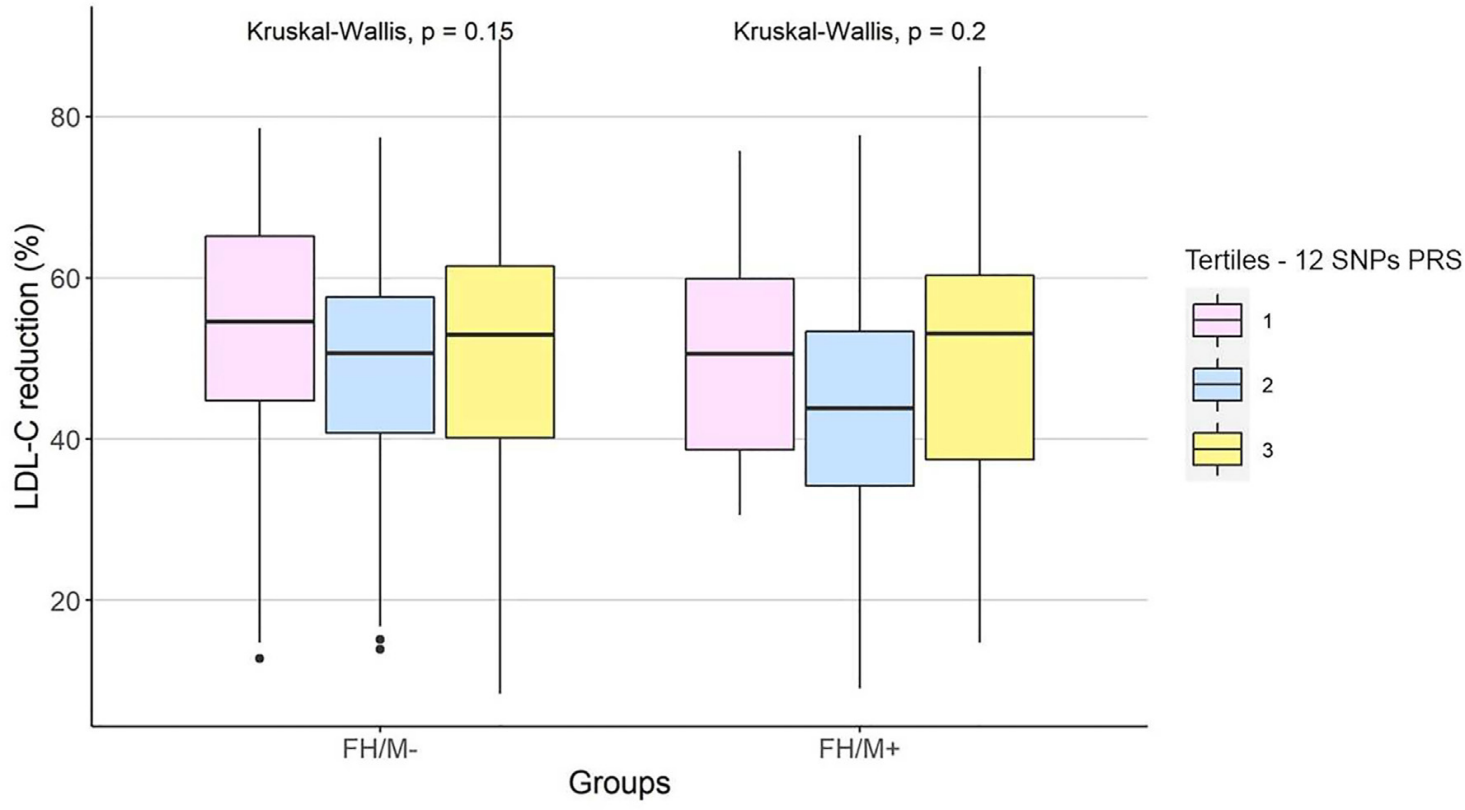
Percentage of LDL-C reduction (LDL-C post treatment minus LDL-C at baseline) after lipid-lowering therapy with statins and/or ezetimibe in the FH groups according to the tertiles of 12 SNPs PRS.

Of the total individuals in the FH/M− group, 226 (46.02%) had pre- and post-pharmacological treatment LDL-C values, with a mean on-treatment LDL-C value of 121 mg/dL. Likewise, 89 patients in the positive group (46.11%) had pre- and post-pharmacological treatment LDL-C values with an average of 145 mg/dL after treatment. We investigated whether the LDL-C percentage reduction depended on the PRSs and found no significant difference in LDL-C reduction between the tertiles of each score in both groups. (Fig. 4 for 12 SNPs PRS and Supplementary Figure 5 for 6 SNPs PRS).

### 3.4 Association of PRSs with clinical and preclinical atherosclerosis and manifestations of CVD

CAC score, used as a proxy of preclinical atherosclerosis, was first divided into four different categories: 0, 1–99, 100–400 and, > 400 units. A CAC score of zero was observed in more than 70% of the subjects of the control group, while in the FH groups this frequency was less than 50%. A moderate and severe level of CAC (>100 and > 400, respectively) was detected in 37% of the FH/M+ group, and in about 17% in the FH/M − group (Supplementary Figure 6). Once CAC distributions were non-normal and right-skewed, we performed a binary logistic regression in the control group to determine which quartile of both PRSs had higher risk of developing CVD, using 100 units as a cutoff. After adjustment for age and sex, the OR for having a CAC score >100 was 1.7 (95% CI: 1.01–2.88, *p* = 0.04) in the 4th quartile of the 12 SNPs PRS when compared to the 1st quartile (Table 3). CAC score comparison between quartiles of PRS was not performed in the FH groups because the number of individuals with CAC score >100 in each quartile of the groups was low.

**Table 3:** Association between the quartiles of the 12 SNPs PRS and CAC score >100 in the ELSA-Brasil control group after adjustment for age and sex.

Variables Quartile	OR	95% CI	p-value
1	Ref	–	–
2	1.45	0.85, 2.48	0.2
3	1.27	0.75, 2.17	0.4
4	1.70	1.01, 2.88	0.047
**OR for the trend**	1.15	0.98, 1,36	0.086
Age	1.14	1.12, 1.17	<0.001
Sex			
Female	Ref	–	
Male	6.00	4.03, 9.13	<0.001

Abbreviations – OR: Odds Ratio; CI: Confidence Interval.

Dependent variable: CAC score > 100. Independent variables: 12 SNPs PRS Quartiles.

Logistic regression model intercept: -10.77, p-value; <0.001.

Myocardial infarction, stroke, and myocardial revascularization were defined as previous cardiovascular disease. CVD occurred in a higher proportion in the FH/M+ than the FH/M− group (28.50% and 19.35%, respectively. *p* = 0.012). When adjusted for age, sex, hypertension and smoking habit, FH/M+ subjects had an overall OR of 2.64 (95% CI: 1.66–4.23, *p* < 0.001) of having CVD disease when compared to FH/M− subjects. However, there was no significant difference in the frequency of CVD events between the FH groups in the quartiles of both scores (12 SNPs PRS: *p* = 0.371; 6 SNPs PRS: *p* = 0.227).

## 4 Discussion

In the present study we analyzed a large Brazilian FH cohort for two LDL-C polygenic risk scores reported by Talmud et al. [10] and observed that the subjects who have an FH phenotype but no identified genetic variants in the FH canonical genes have a higher mean PRS, followed by individuals who have a positive genetic diagnosis. Thus, suggesting that hypercholesterolemia in some individuals may have a polygenic rather than a monogenic cause. Moreover, even in the FH/M+ group, elevated LDL-C may have an additional polygenic component, once this group also showed significantly higher PRSs than controls. Our findings are similar to the ones from other studies in individuals with different ancestries [10,22]. Of notice, in our work, PRS was associated with LDL-C concentrations in the Brazilian general population as well as preclinical manifestations of atherosclerosis. However, in people with the FH phenotype no association was encountered with the latter and with response to lipid lowering pharmacological therapy. Our results extend these findings for a Latin American population, as well as, for a new range of clinically related traits in FH.

Both scores showed a moderate association with LDL-C in the control group. In this cohort, the linear regression coefficient showed that those models were able to explain about 4% of the overall LDL-C variability. Possibly, the allele frequency among different ethnics groups explains why this value was lower than the observed in a British cohort, where the regression model explained about 11% of this variability [10], but higher than the observed in a Korean cohort, where about 2% of LDL-C was explained by the regression model [14]. Naturally, the major limitation of this study is the use of SNPs identified using mainly European samples. Gratton et al. (2022) [15] demonstrated that this PRS had better performance in individuals with UK Black and Caribbean and White ancestries than Asians individuals and proposed the hypothesis of the adoption of a specific-ethnic decile cut-off value than a general one. Likewise, as shown by Toft-Nielsen and colleagues [23], FH prevalence diverge among different ancestries, being more prevalent in Black and White than Asian individuals.

Additionally, it is necessary to assess the need to develop more accurate PRSs, using different sets of SNPs. Wu and colleagues [24] developed a PRS that explained 21% of LDL-C variability in a Caucasian British population. Nonetheless, when validating the score in non-Caucasian individuals, the predictive performance of the score decreased, indicating that PRSs must be calibrated when applied to different ancestries. Although we have used the effect estimates originally described, we also determined the β-coefficients using individuals of the control group for comparison (Supplementary Table 4). Vrablik et al. [25] showed that the impact of SNPs associated with lipids fractions detected by GWAS may diverge in different populations, where the same variant can have different effects on the phenotype. However, we did not observe major differences in minor allele frequencies values among our population and others (Supplementary Table 5).

We did not observe LDL-C difference between the tertiles of both scores in individuals in the FH groups but only in the control population. Unlike our research, most studies that assess the applicability of these scores use cohorts with a milder FH phenotype, with LDL-C around 190 mg/dL or following the DLNC or Simon Broome criteria [10,13,26]. The stricter inclusion criterion of LDL-C >210 mg/dL may have disfavored the LDL-C association with the score tertiles, in contrast to the current literature.

Mickiewicz et al. [27] showed that individuals with polygenic dyslipidemia had a better response to low doses of statin than individuals with monogenic dyslipidemia. Furthermore, other studies showed that the presence and type of genetic variant directly influenced LDL-C reduction since FH/M − individuals had a greater response to statins [28,29]. When investigating if lipid-lowering therapy efficiency was associated with the PRSs, we did not observe any correlation between LDL-C reduction with treatment and PRSs regardless of the presence or absence of a genetic mutation or the baseline LDL-C value.

Multiple studies have shown that CAC score of zero indicates low CVD risk, while CAC >100 is associated with a significant increase in event rate even in populations with FH, being a good surrogate of the severity of preclinical atherosclerosis [30,31]. In the present study, we have shown that subjects of the general population in the 4th quartile of the 12 SNPs PRS had a 74% increased odds of having CAC > 100 and consequently, a greater chance of clinical atherosclerotic events. However, CAC data were not available for all individuals in the FH group, being thus a limitation in the current analysis.

The risk of developing atherosclerosis and of having a CVD event has also been shown to be proportional to the long-term exposure to high LDL-C values and the presence of pathogenic genetic variation, as previously shown by our group and others [[32], [33], [34]]. Since the FH/M+ group had a higher mean LDL-C and an expectedly higher cumulative exposure, we could also expect that this group would have an increased frequency of CVD than the FH/M− , as shown in this study. Trinder et al. [26] pointed that subjects who had only elevated FH polygenic risk score had a similar risk than patients with no FH-causing variant and a low polygenic score, but patients with both monogenic FH and high polygenic score had the highest risk of premature CVD when compared to subjects who have only a monogenic cause. Likewise, other groups also demonstrated a higher risk of CVD in individuals with high PRS, confirming the polygenic contribution to the phenotypes and its clinical implications [35,36]. However, we did not observe a significant association between PRS and higher odds of CVD, or subclinical atherosclerosis, within each FH group. Probably the reduced statistical power due to the limited sample size hindered our ability to identify a predictive role for LDL-C PRS in this specific clinical scenario. Nonetheless, a PRS specific for CVD, as opposed to only LDL-C, could be more useful for cardiovascular risk stratification in FH as previously suggested and ascertained [35,37].

Further studies are necessary to validate PRSs in the Latin American FH populations and to clarify why a proportion of patients in the FH/M− group, even having high values of LDL-C, still do not have either a monogenic or polygenic cause identified. The availability of numerous GWAS can allow the improvement of a more comprehensive PRS, with greater risk stratification power and applicability [38]. Also, some other genes may be involved in the genetics of FH, such as *APOE*, *STAP1* and, *LIPA,* but the exact contribution of those genes remains unclear [39]. In the present study, 35% of the subjects in the two FH groups had a complete screening of those genes, excluding this possibility as an explanation for the low yield of molecular testing in FH. On the contrary, our results support the proposition that a significant fraction of people with the FH phenotype are, indeed, individuals with polygenic hypercholesterolemia.

The main study limitations are its cross-sectional design, the small number of individuals with the FH phenotype and CAC scores; the use of a subsample of participants from the ELSA-Brasil study and not the whole cohort; and the replacement in NGS technology, once not all subjects were sequenced using the same panel. Furthermore, the lack of information about the type, dosage, and duration of pharmacological treatment in the FH groups is also a limitation of the study.

We conclude that this is the first study to evaluate two PRSs associated with dyslipidemia as a risk assessment tool in a Latin American sample of FH individuals. Our study showed that subjects with the FH phenotype, but in whom no causal genetic variant was identified had a higher mean PRS when compared to FH/M+ and control groups. In addition, despite not predicting LDL-C levels, CVD events or atherosclerosis in the FH population, the 12 SNPs PRS has been shown to be a risk predictor of preclinical atherosclerosis in the general population.

## Financial Support

IRL has received a scholarship from Fundação Coordenação de Aperfeiçoamento de Pessoal de Nível Superior, Brazil (CAPES) #88887.531079/2020-00. RDS is recipient of a scholarship from Conselho Nacional de Pesquisa e Desenvolvimento Tecnológico, Brazil (CNPq) #303734/2018-3. The ELSA-Brasil baseline study was supported by the 10.13039/501100006506Brazilian Ministry of Health (Science and Technology Department) and the BrazilianMinistry of Science and Technology (10.13039/501100004809Financiadora de Estudos e Projetos and 10.13039/501100003593CNPq 10.13039/100013101National Research Council). The funding of Sociedade Hospital Samaritano and Ministério da Saúde (PROADI-SUS; SIPAR: 25000.180.672/2011-81) are gratefully acknowledged.

## Author contributions

IRL conceptualized and designed the study, researched data, performed experiments and wrote the manuscript. IRL and MTT were responsible for carrying out the genetic tests, analyzed and interpreted the results. ACP and TGMO assisted with the data analysis. ACP and CEJ were responsible for the HipercolBrasil program and assisted with the data collection. IB and PA were responsible for the ELSA-Brasil program and assisted with the data collection. RDS gave clinical support, acquired data and drafted the manuscript. ACP and JEK supervised the work. All authors reviewed the manuscript.

## Declaration of competing interest

The authors declare the following financial interests/personal relationships which may be considered as potential competing interests: RDS has received honoraria related to consulting, research and or speaker activities from: Abbott, Amgen, Aché, Astra Zeneca, Esperion, EMS, GETZ Pharma, Kowa, Libbs, Merck, MSD, Novo-Nordisk, Novartis, PTC Therapeutics, Pfizer, Roche and Sanofi.

IRL has received a scholarship from Fundação Coordenação de Aperfeiçoamento de Pessoal de Nível Superior, Brazil (CAPES) #88887.531079/2020-00. RDS is recipient of a scholarship from Conselho Nacional de Pesquisa e Desenvolvimento Tecnológico, Brazil (CNPq) #303734/2018-3. The ELSA-Brasil baseline study was supported by the 10.13039/501100006506Brazilian Ministry of Health (Science and Technology Department) and the BrazilianMinistry of Science and Technology (10.13039/501100004809Financiadora de Estudos e Projetos and 10.13039/501100003593CNPq 10.13039/100013101National Research Council).
