# Supplementary material for: Polygenic risk score for hypercholesterolemia in a Brazilian familial hypercholesterolemia cohort
Source: Atheroscler Plus. 2022 Jun 28;49:47–55. doi: 10.1016/j.athplu.2022.06.002 (PMC9833269; doi:10.1016/j.athplu.2022.06.002)
Supplement: Multimedia component 1 [file mmc1.docx]

**SUPPLEMENTARY MATERIAL**

**Supplementary Table 1:** Difference between mean LDL-C value in each tertile of scores according to ancestry in the ELSA-Brasil control group.

|  | |  | | **ANCESTRY GROUPS** | | **LDL-C (mean)** | ***p* value** | ***p* adj value** | |
| --- | --- | --- | --- | --- | --- | --- | --- | --- | --- |
| **TERTILES –**  **12 SNPs PRS** | | **1** | | White vs. Black | 117.72 and 122.32 | 0.272 | | 1 |  |
|  |  |  |  | White vs. Mixed | 117.72 and 124.22 | 0.05 | | 0.4 |  |
|  |  |  |  | Black vs. Mixed | 122.32 and 124.22 | 0.613 | | 1 |  |
|  |  | **2** | | White vs. Black | 131.90 and 134.80 | 0.405 | | 1 |  |
|  |  |  |  | White vs. Mixed | 131.90 and 135.14 | 0.788 | | 1 |  |
|  |  |  |  | Black vs. Mixed | 134.80 and 135.14 | 0.596 | | 1 |  |
|  |  | **3** | | White vs. Black | 133.30 and 145.18 | 0.008 | | 0.077 |  |
|  |  |  |  | White vs. Mixed | 133.30 and 137.98 | 0.069 | | 0.48 |  |
|  |  |  |  | Black vs. Mixed | 145.18 and 137.98 | 0.273 | | 1 |  |
| **TERTILES**  **- 6 SNPs PRS** | | **1** | | White vs. Black | 118.15 and 121.68 | 0.384 | | 1 |  |
|  |  |  |  | White vs. Mixed | 118.15 and 125.40 | 0.038 | | 0.3 |  |
|  |  |  |  | Black vs. Mixed | 121.68 ans 125.40 | 0.407 | | 1 |  |
|  |  | **2** | | White vs. Black | 131.55 and 133.95 | 0.402 | | 1 |  |
|  |  |  |  | White vs. Mixed | 131.55 and 132.08 | 0.839 | | 1 |  |
|  |  |  |  | Black vs. Mixed | 133.95 and 132.08 | 0.561 | | 1 |  |
|  |  | **3** | | White vs. Black | 133.25 and 146.82 | 0.004 | | **0.04** |  |
|  |  |  |  | White vs. Mixed | 133.25 and 139.65 | 0.109 | | 0.76 |  |
|  |  |  |  | Black vs. Mixed | 146.82 and 139.65 | 0.112 | | 0.76 |  |

*P*-value adjusted with Holm–Bonferroni method for multiple comparisons.

| **Supplementary Table 2:** Comparative means of the polygenic risk scores between groups. | | | | |
| --- | --- | --- | --- | --- |
|  | **12 SNPs PRS** | | **6 SNPs PRS** | |
|  | **Mean (SD)** | ***p* value** | **Mean (SD)** | ***p* value** |
| **ELSA-Brasil vs. FH/M-** | 33.10 (9.13) and 38.25 (7.23) | 2.2x10^-16^ | 23.03 (8.80) and 27.82 (6.87) | 2.2x10^-16^ |
| **ELSA-Brasil vs. FH/M+** | 33.10 (9.13) and 36.48 (6.97) | 4.3x10^-7^ | 23.03 (8.80) and 26.26 (6.66) | 2.7x10^-6^ |
| **FH/M- vs. FH/M+** | 38.25 (7.23) and 36.48 (6.97) | 0.001 | 27.82 (6.87) and 26.26 (6.66) | 0.001 |

**Supplementary Table 3:** Comparative analysis of both scores among whites and non-whites.

| **Ancestry** | **Group** | **12 SNPs PRS** | | **6 SNPs PRS** | |
| --- | --- | --- | --- | --- | --- |
|  |  | **Mean (SD)** | ***p* value** | **Mean (SD)** | ***p* value** |
| **Non-white** | ELSA-Brasil  (n = 629) | 31.56 (9.31) | < 0.001 | 21.91 (8.96) | < 0.001 |
|  | FH  (n = 186) | 37.61 (7.43) |  | 27.32 (6.94) |  |
| **White** | ELSA-Brasil  (n = 959) | 34.10 (8.90) | < 0.001 | 23.76 (8.65) | < 0.001 |
|  | FH  (n = 389) | 38.01 (6.82) |  | 27.56 (6.49) |  |

**Supplementary Table 4:** Comparison of the beta-coefficients (GLGC and ELSA-Brasil).

| **SNP** | **Gene** | **Minor Allele** | **Common Allele** | **GLGC weight (mg/dL)** | **ELSA weight**  **(mg/dL)** |
| --- | --- | --- | --- | --- | --- |
| rs2479409 | *PCSK9* | **G** | A | 2.01 | 0.287 |
| rs629301 | *CELRS2* | G | **T** | 5.65 | 5.271 |
| rs1367117 | *APOB* | **A** | G | 4.05 | 3.028 |
| rs4299376 | *ABCG8* | **G** | T | 2.75 | 2.779 |
| rs1564348 | *SLC22A1* | C | **T** | 0.56 | -4.670 |
| rs1800562 | *HFE* | A | **G** | 2.22 | 5.715 |
| rs3757354 | *MYLIP* | T | **C** | 1.43 | 1.904 |
| rs11220462 | *ST3GAL4* | **A** | G | 1.95 | 2.951 |
| rs8017377 | *NYNRIN* | **A** | G | 1.14 | 0.129 |
| rs6511720 | *LDLR* | T | **G** | 6.99 | 5.328 |
| rs429358 | *APOE* | C | T | - | - |
| rs7412 | *APOE* | T | C | - | - |
| ɛ2ɛ2 | *APOE* |  |  | -34.75 | -25.47 |
| ɛ2ɛ3 | *APOE* |  |  | -15.45 | -17.58 |
| ɛ2ɛ4 | *APOE* |  |  | -7.72 | -3.43 |
| ɛ3ɛ3 | *APOE* |  |  | 0 | 0 |
| ɛ3ɛ4 | *APOE* |  |  | 3.86 | 4.06 |
| ɛ4ɛ4 | *APOE* |  |  | 7.72 | -2.50 |

**Supplementary Table 5:** Minor-Allele frequencies of the SNPs presented in the scores in different populations.

| **SNP** | Gene | Minor Allele | Common Allele | MAF ABraOM  (n = 1171) | MAF GnomAD  (n = 141456) | MAF ELSA-Brasil  ( n = 1605) | MAF FH/M+  (n = 193) | MAF FH/M-  (n = 491) |
| --- | --- | --- | --- | --- | --- | --- | --- | --- |
| rs2479409 | *PCSK9* | G* | A | 0.396 | 0.344 | 0.358 | 0.388 | 0.400 |
| rs629301 | *CELRS2* | G | T* | 0.251 | 0.249 | 0.244 | 0.166 | 0.180 |
| rs1367117 | *APOB* | A* | G | 0.255 | 0.256 | 0.241 | 0.285 | 0.317 |
| rs4299376 | *ABCG8* | G* | T | 0.271 | 0.243 | 0.271 | 0.290 | 0.311 |
| rs1564348 | *SLC22A1* | C | T* | 0.142 | 0.138 | 0.163 | 0.184 | 0.178 |
| rs1800562 | *HFE* | A | G* | 0.020 | 0.034 | 0.020 | 0.020 | 0.014 |
| rs3757354 | *MYLIP* | T | C* | 0.219 | 0.283 | 0.246 | 0.280 | 0.224 |
| rs11220462 | *ST3GAL4* | A* | G | 0.103 | 0.131 | 0.101 | 0.109 | 0.120 |
| rs8017377 | *NYNRIN* | A* | G | 0.342 | 0.363 | 0.348 | 0.399 | 0.373 |
| rs6511720 | *LDLR* | T | G* | 0.125 | 0.111 | 0.134 | 0.111 | 0.058 |
| rs429358 | *APOE* | C | T | 0.130 | 0.142 | 0.139 | 0.153 | 0.215 |
| rs7412 | *APOE* | T | C | 0.062 | 0.065 | 0.070 | 0.023 | 0.018 |

Abbreviations - MAF: Minor-Allele Frequency; SNP: Single Nucleotide Variant; ABraOM: Arquivo Brasileiro Online de Mutações (Online Archive of Brazilian Mutations); GnomAD: Genome Aggregation Database

* Risk allele used to calculate both PRSs.


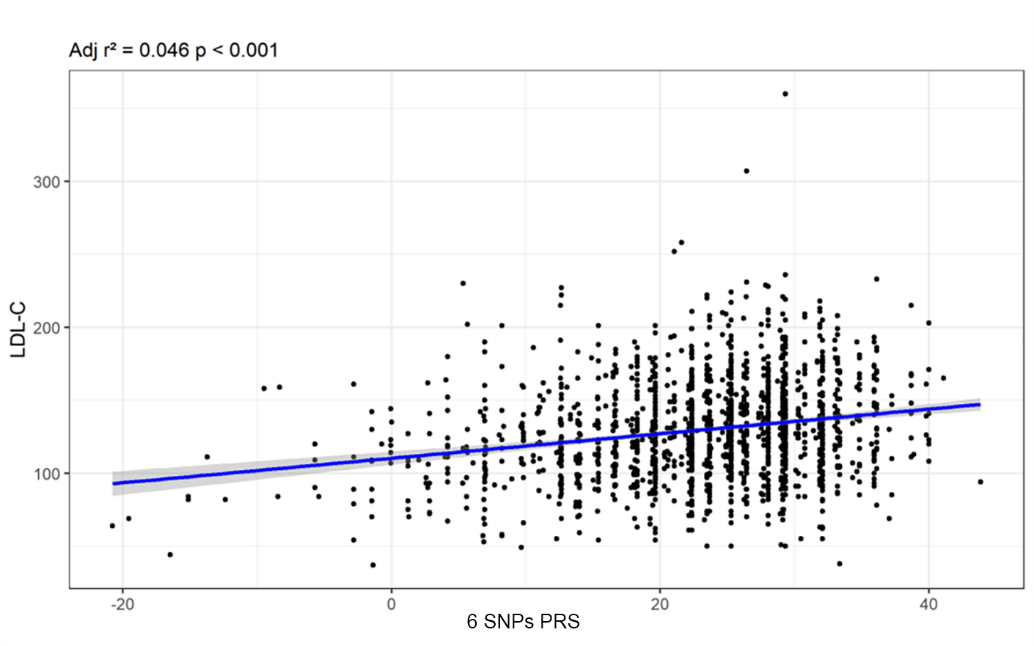


**Supplementary Figure 1**: Association between 6 SNPs PRS and LDL-C in individuals from the ELSA-Brasil group.


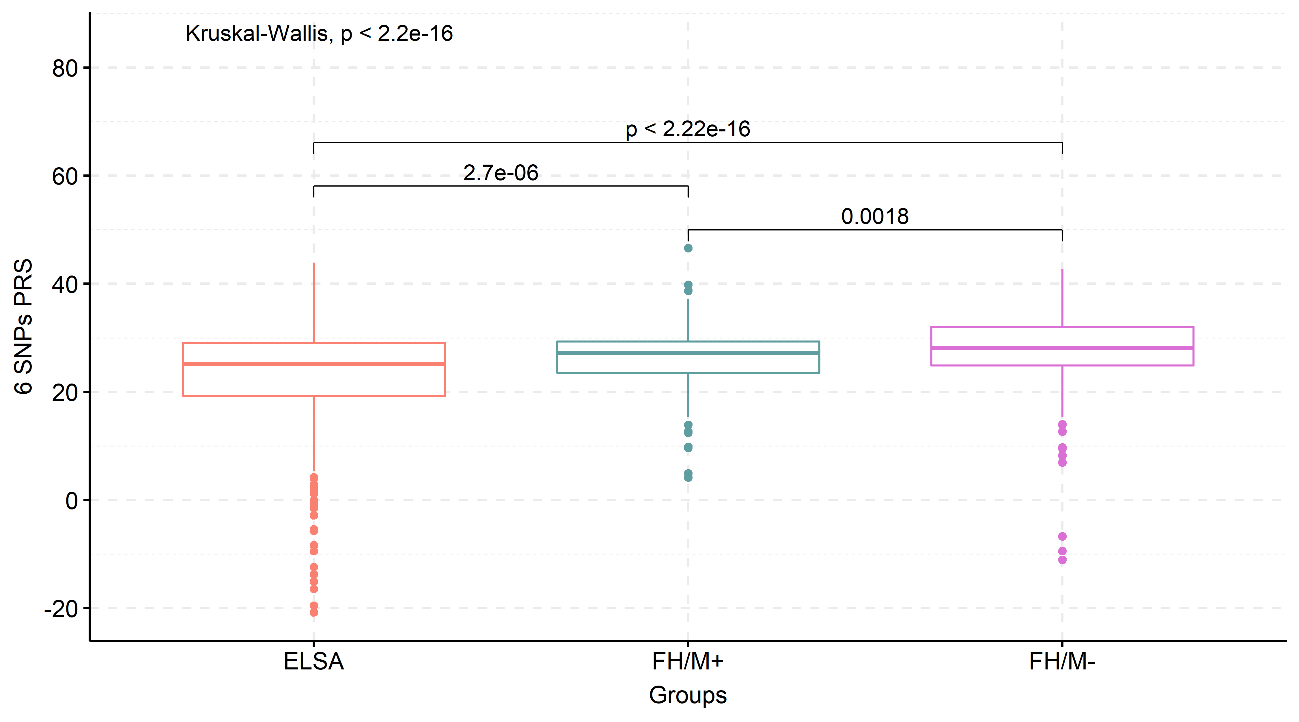


**Supplementary Figure 2:** Comparison of mean 6 SNPs PRS between groups


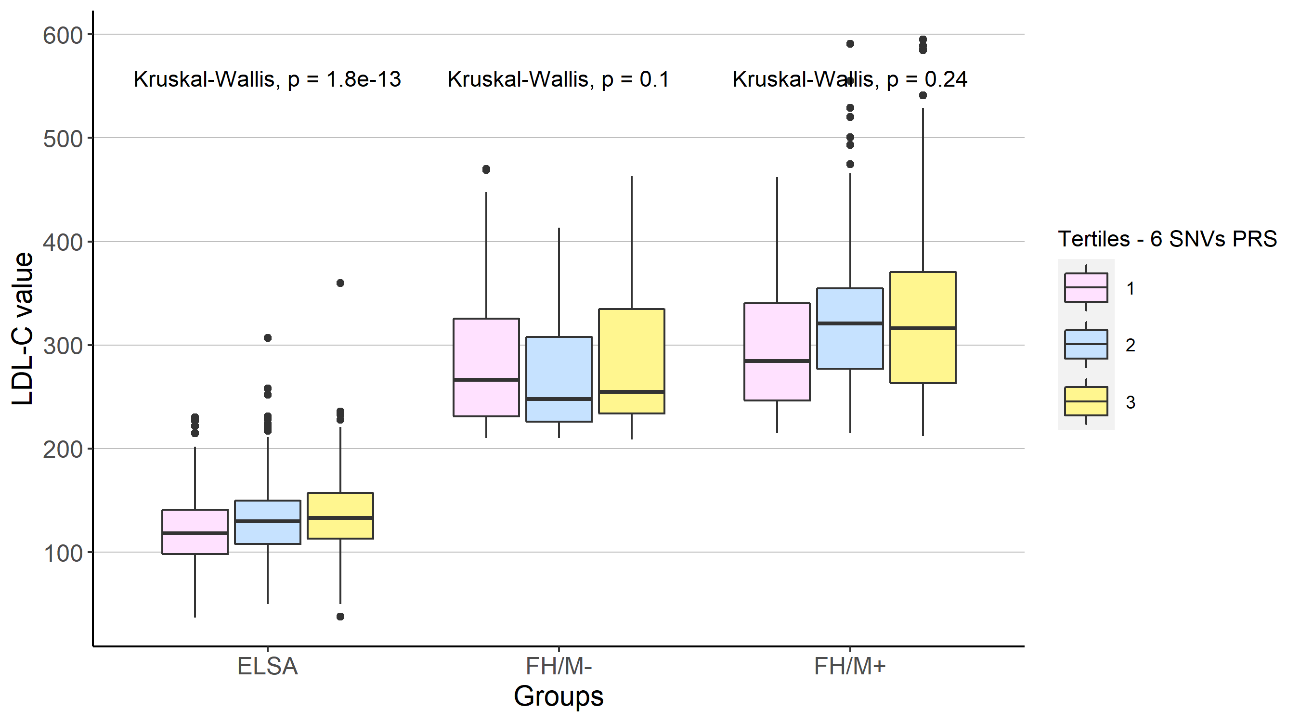


**Supplementary Figure 3:** Distribution of LDL-C value between tertiles of 6 SNPs PRSs in each study group.


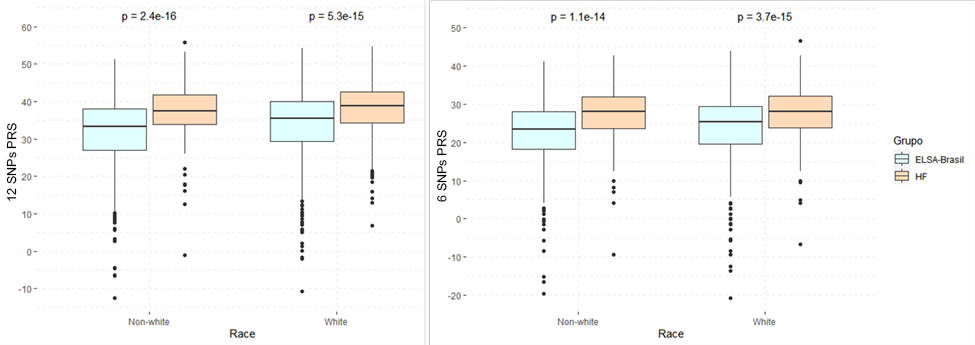


**Supplementary Figure 4:** Distribution of weighted 12 SNPs PRS and 6 SNPs PRS among white and non-whites.


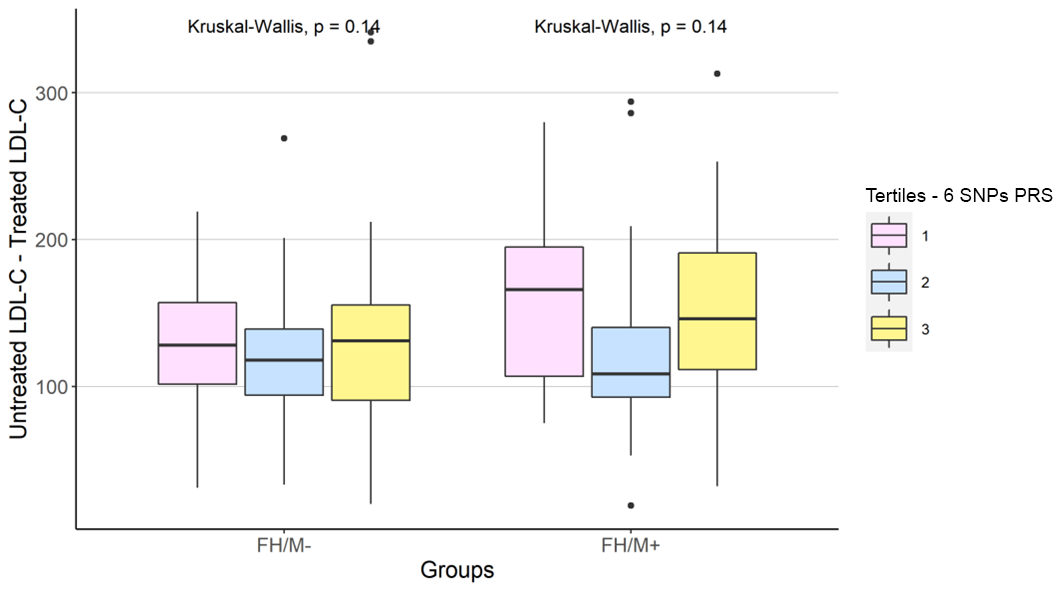


**Supplementary Figure 5:** LDL-C reduction (LDL-C untreated - LDL-C treated) in the FH groups in the tertiles of 12 SNPs PRS.

***
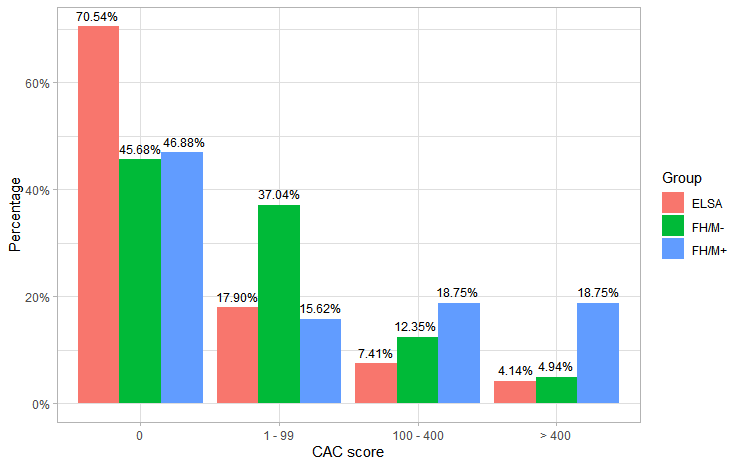
***

**Supplementary Figure 6:** Percentage of individuals in each group in the CAC score categories

**
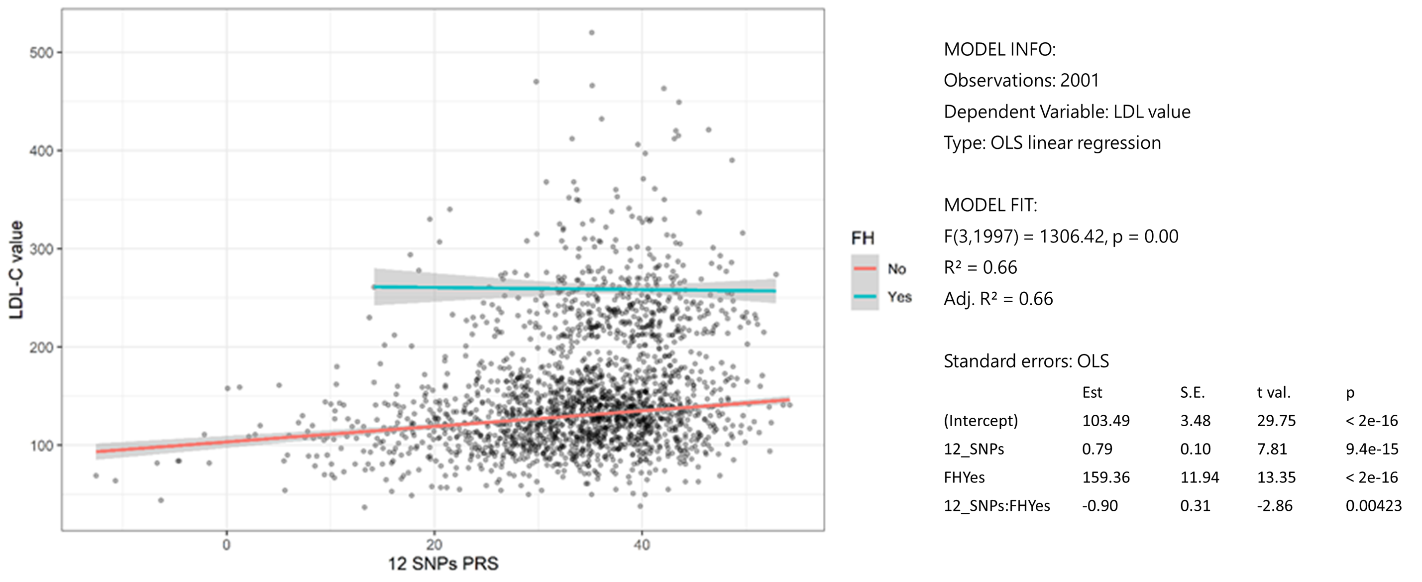

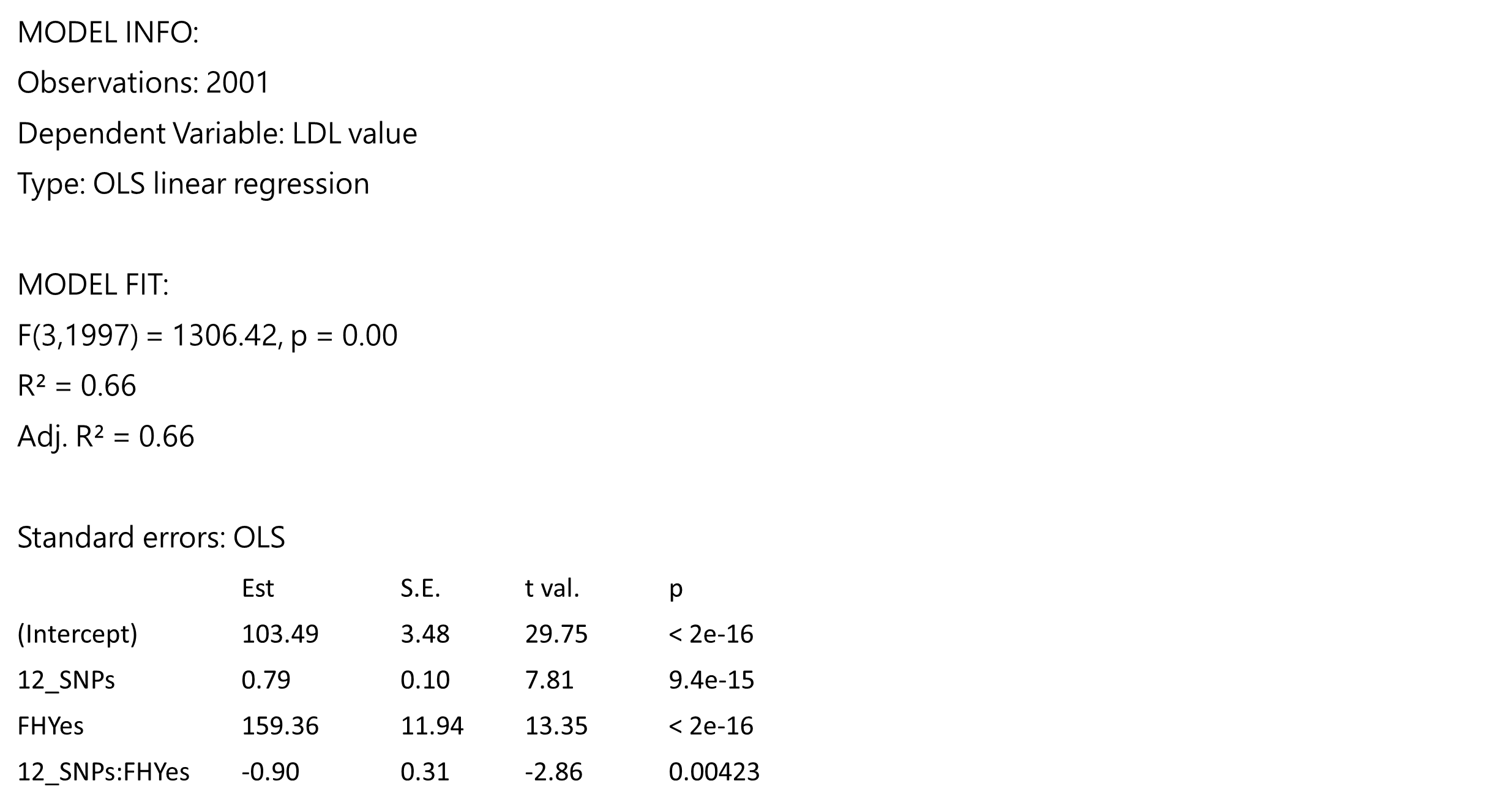
**

**Supplementary Figure 7:** Association between 12 SNPs PRS and LDL-C in the individuals in the study computing the statistical interaction PRS x FH according to the presence or absence of FH.


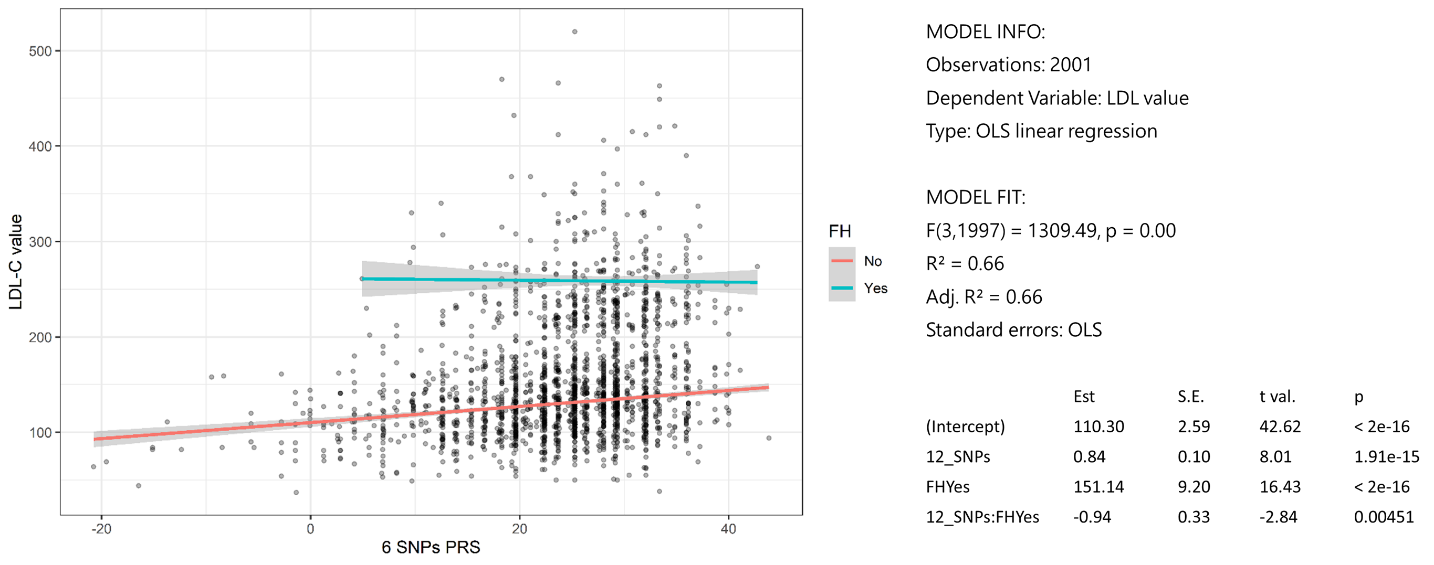


**Supplementary Figure 8:** Association between 6 SNPs PRS and LDL-C in the individuals in the study computing the statistical interaction PRS x FH according to the presence or absence of FH.


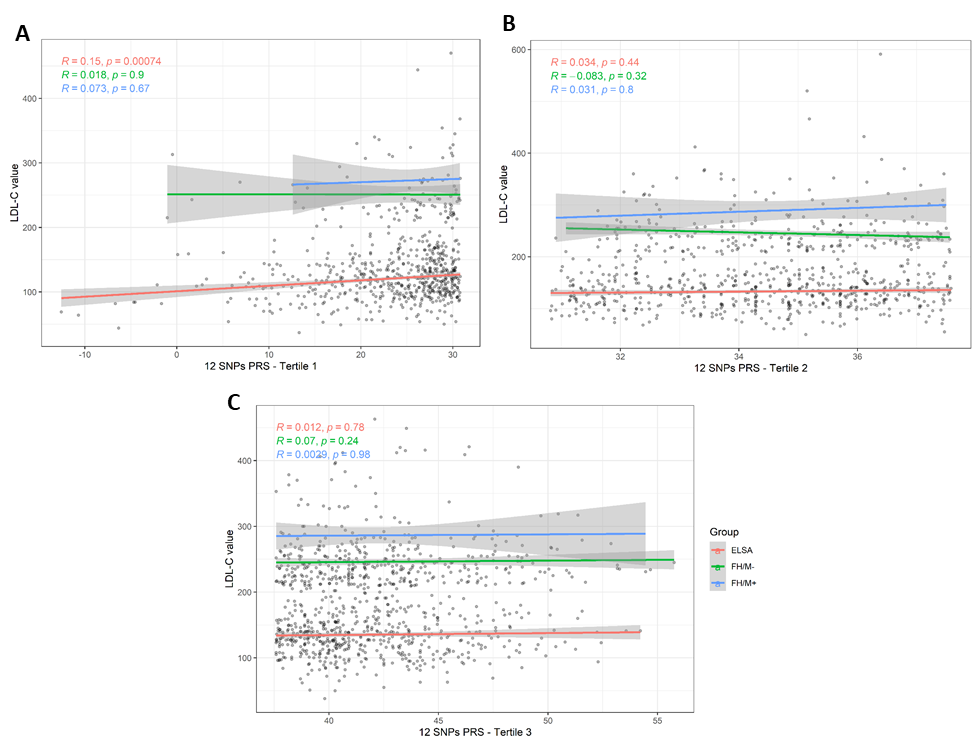


**Supplementary Figure 9:** Spearman correlation between LDL-C and PRS across the tertiles of the 12 SNPs PRS. **A -** Tertile 1; **B -** Tertile 2; **C -** Tertile 3
